# Cross-Modality Information Transfer: A Hypothesis about the Relationship among Prehistoric Cave Paintings, Symbolic Thinking, and the Emergence of Language

**DOI:** 10.3389/fpsyg.2018.00115

**Published:** 2018-02-20

**Authors:** Shigeru Miyagawa, Cora Lesure, Vitor A. Nóbrega

**Affiliations:** ^1^Department of Linguistics and Philosophy, Massachusetts Institute of Technology, Cambridge, MA, United States; ^2^Center for Research and Development of Higher Education, The University of Tokyo, Tokyo, Japan; ^3^Department of Linguistics, University of São Paulo, São Paulo, Brazil

**Keywords:** symbolic thinking, language, cave art, archeoacoustics, Khoisan

## Abstract

Early modern humans developed mental capabilities that were immeasurably greater than those of non-human primates. We see this in the rapid innovation in tool making, the development of complex language, and the creation of sophisticated art forms, none of which we find in our closest relatives. While we can readily observe the results of this high-order cognitive capacity, it is difficult to see how it could have developed. We take up the topic of cave art and archeoacoustics, particularly the discovery that cave art is often closely connected to the acoustic properties of the cave chambers in which it is found. Apparently, early modern humans were able to detect the way sound reverberated in these chambers, and they painted artwork on surfaces that were acoustic “hot spots,” i.e., suitable for generating echoes. We argue that cave art is a form of cross-modality information transfer, in which acoustic signals are transformed into symbolic visual representations. This form of information transfer across modalities is an instance of how the symbolic mind of early modern humans was taking shape into concrete, externalized language. We also suggest that the earliest rock art found in Africa may constitute one of the first fossilized proxies for the expression of full-fledged human linguistic behavior.

## Introduction

An extraordinary trait that humans have, one that separates us from all other living beings, is our “unique symbolic cognitive style” (Tattersall, 2017). As the philosopher Ernst Cassirer noted, humans are not the *animal rationale* but the *animal symbolicum* (Cassirer, 2006, p. 31). Although other animals are capable of challenging cognitive behavior — for instance, the crow’s ability to make stick tools (Bluff et al., 2007), and the apparent symbolically mediated behavior of late Neanderthal populations (Jaubert et al., 2016) — the human capacity for symbolic thinking is immeasurably greater and qualitatively distinct, so much so that Charles Darwin himself commented, “the difference between the mind of the lowest man and that of the highest animal is immense” (Darwin, 1871, p. 100). Alfred Russel Wallace, a co-discoverer of evolution by natural selection, was particularly puzzled because he did not see tangible evolutionary advantages to the products of this unique capacity for symbolic thinking, such as music and the arts (Wallace, 1870). Assuming that early human symbolic behavior can be read from the archeological record, we explore the emergence of cave and rock art in human evolution and assess its relation to the development of human language.

## When Did Symbolic Thinking Appear?

When did we acquire this cognitive capacity for symbolic thinking?^1^ The answer to this question must necessarily be based on indirect evidence, since we do not have access to facts about the variability and heritability of this trait (Lewontin, 1998). Suppose we equate high cognitive ability with brain size. The hominid brain has been growing for 2 million years, doubling in size twice during that period (Holloway et al., 2004), with modern humans at the end of the line showing the highest encephalization quotient (Finlay, 2009). However, Neanderthals had a brain that was larger in volume than humans (Holloway, 1981). Even after discounting for the disproportionate size of the area dedicated to visual perception (Pearce et al., 2013), their brain size was still comparable to that of modern humans. Yet they did not develop the kinds of behavior, such as agriculture and language, that we associate with high cognitive ability (Tattersall, 2008, 2010).

Is there something else in human evolutionary history that would indicate some drastic and qualitative change in behavior, signaling the emergence of symbolic thinking? Tattersall (2008, 2012, 2016a,b, 2017) makes an intriguing observation about the pace of technological innovation. The first stone-tool technology appeared 2.5 million years ago (Semaw et al., 1997), and it stayed basically the same for a million years before innovation was introduced in the form of the Acheulean handaxe. Another million years went by before a significant innovation took place, in the form of core preparation. In other words, innovation was rare and interspersed with long stretches during which hardly any change occurred. But toward the end of the Pleistocene, a profound shift occurred: technological innovations began to appear in rapid progression, and this marked a “relatively abrupt and qualitative change in mental information processing” (Tattersall, 2017, p. 5). This era of rapid change corresponds approximately to markers of symbolic thinking, such as the pieces of engraved ochres (Henshilwood et al., 2002) and the marine pierced shells (Henshilwood et al., 2004; d’Errico et al., 2005) found at Blombos Cave, which have been dated back to around 70,000–100,000 years ago.^2^ These constitute the first archeological proxies signaling the fixation of the human language faculty (Huijbregts, 2017).

## Beginnings of Symbolic Thinking

**FIGURE 1:**
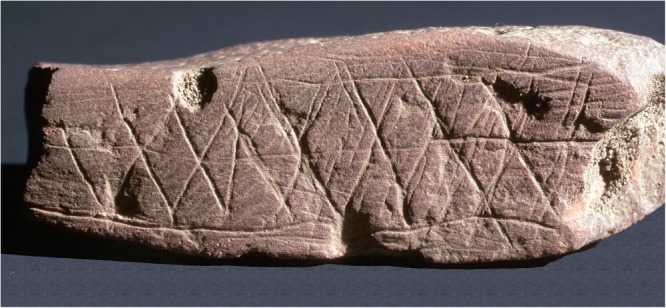
Ochres from the Blombos Cave (Evolutionary Studies Institute University of the Witwatersrand).

An often-noted early piece of evidence for symbolic thinking is the two slabs of ochre from the Blombos Cave in South Africa. Ochre is an iron-rich mineral that served several roles, including body decoration, along with more utilitarian roles (Watts, 2009; Hansen, 2011). In Blombos Cave, more than 8,000 pieces of ochre-like material have been found (Rosso et al., 2017). Some, like those in **Figure 1**, have geometric engravings and incisions.

It has been suggested that these regular patterns are a proxy for symbolic thinking (Henshilwood et al., 2002; Tattersall, 2009).^3^ The idea is that the ochre engravings are an external, abstract representation of internal high-order cognitive processes. This is similar to spoken language, which is an external form of highly complex internal cognitive representations and computation. Although Neanderthals produced etchings (Rodríguez-Vidal et al., 2014) and geometric structures (Jaubert et al., 2016), they apparently did not possess the cognitive capabilities that modern humans do (Tattersall, 2008, 2010; Mendez et al., 2016; Sankararaman et al., 2016; Vernot et al., 2016).^4^ The mechanisms for this heightened cognition involve computational processes that may also occur in other animals but that in humans “are uniquely powerful in their range, capacity and flexibility” (Heyes, 2012).^5^

## Life in the Caves

The rapid innovation in tool making and the production of the Blombos Cave ochres and pierced shells suggest that modern humans by around 100,000 years ago were able to tap some cognitive resource that had not existed before. We will look at a well-known phenomenon that heretofore has been sparsely considered as exemplifying symbolic thinking. This is the phenomenon of cave and rock art, which is found on every major continent occupied by modern humans (Bahn and Fossati, 1995, 2003; Bahn et al., 2008, 2012). We wish to understand the nature of the expressions of symbolic thinking inherent in these artifacts as a way to begin to understand the evolutionary process that led to a fully developed symbolic species. We will show that our findings about these artifacts parallel aspects of human language.

We propose that the phenomenon of cave and rock art plausibly indicates how an internalized “system of thought” (Chomsky, 2011, 2013, 2015, 2016), which presumably evolved with the speciation of modern *Homo sapiens* around 200,000 years ago (Huijbregts, 2017), may have taken shape into concrete, externalized language. If this turns out to be true, the often-stated idea that “language does not fossilize” (e.g., Deacon, 1997; Fitch, 2010; Berwick and Chomsky, 2016) is not quite true: pieces of externalized language may turn out to be hidden among the art forms produced by our early modern human ancestors.

Some of the most striking artifacts from the life of early humans are the art forms found in caves throughout the world. Some of the most well-known are the Upper Paleolithic examples found in France and Spain. There are a number of puzzling features of this cave art that until recently escaped any rational explanation. These pictographs and petroglyphs are often found deep inside a cave, frequently in inaccessible locations. They tend to cluster narrowly in one location, ignoring nearby surfaces that appear to be just as suitable. And over ninety percent of the figures consist of hoofed animals (Gourhan-Leroi, 1967, 1982; Waller, 1993a, 2006).

**FIGURE 2:**
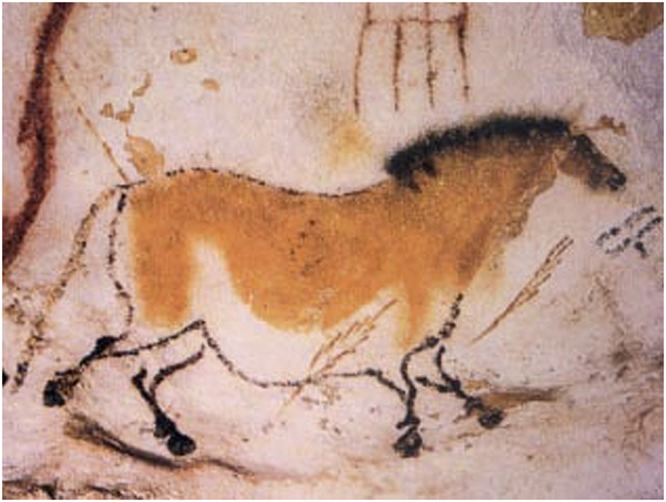
Cave painting of a dun horse from Lascaux, circa 15,000 BC (Wikipedia)^9^.

A subfield of archeology, called archeoacoustics, has produced the idea that cave paintings are intimately related to the acoustic nature of the cave chambers (Reznikoff, 1987; Reznikoff and Dauvois, 1988).^6^ Hoffman (2014), for example, used a laptop and loudspeakers “to sweep a sine wave tone through all audio frequencies, recording the results to capture the acoustic fingerprint of each space.” Such detailed studies of prehistoric sites support the idea that the subject matter and location of the pictures relate directly to the acoustics of the cave structure. Waller (2002) points out that the pictures often cluster in areas with enhanced acoustic properties. For instance, in the deep caves of Font-de-Gaume and Lascaux, pictures of hoofed animals such as bulls, bison, and deer appear in chambers in which the echoes, resonances, and reverberation created percussive sounds that resemble hoof beats^7^, as illustrated in **Figure 2**. In contrast to this, in chambers that are acoustically quiet, one finds pictures of felines (Waller, 1993a) or simple dots and handprints (Hoffman, 2014).^8^

Thus, acoustics offers a compelling explanation for the location of paintings in chambers deep inside caves, because these chambers have special acoustic properties; the clustering of paintings in certain areas of the cave wall, because they are acoustic “hot spots” (Blesser and Salter, 2009; Mattioli et al., 2017); and the predominance of hoofed animals as the subject matter of the paintings. Additionally, stalagmites and stalactites that ring like a musical instrument when struck have been found to be marked with paint (Hoffman, 2014).

### Cross-Modality Information Transfer

Cave art, as analyzed by archeoacoustics, shows a flow of information from one modality to another: auditory to visual. The auditory modality is triggered by external input—thunder, rock tapping, music— and the auditory representation is mentally transformed into external, visual representation. This is a pure form of externalized symbolic thinking where information from one modality is transformed into representation in another modality. We speculate that this activity of information transfer across modalities allowed early humans to enhance their ability to convey symbolic thinking to their conspecifics, as well as their ability to process acoustic and visual input as symbolic (i.e., to associate acoustic and visual stimuli to a given mental representation).

Based on the archeological record we just reviewed, the externalization of the symbolic mode of thinking occurred some 100,000 years ago. It is possible that the cognitive underpinnings of symbolic thinking appeared at the time of the major genetic reorganization that resulted in the physical entity *H. sapiens* (Henshilwood and Dubreuil, 2009), and externalization occurred much later (Chomsky, 2010, 2013, 2017; Huijbregts, 2017). Or externalization may have begun closer to the formation of the new cognitive capacity. The activity of cross-modality information transfer (CMIT) constitutes one major effort to connect the internalized system of thought to sensorimotor systems capable of representing and processing acoustic and visual stimuli.^10^

The idea that an activity like cave art, a form of CMIT, could have had such an enormous consequence for the development of modern humans is plausible on a couple of grounds. First, enormous effort was expended over 1000s of years to create this art (Waller, 2006). We hypothesize that the individuals who were able to transform symbolic thinking into sensory stimuli —likely privileged in the society— may have had a higher rate of reproductive success, thus spreading the cognitive ability required for this practice through the population.^11^

Second, the population of *H. sapiens* early on was compact, around 9,000 when it emerged in Africa (Gronau et al., 2011). A cognitive modification even among a small group —viz., the cave artists— could have cut a large swath through the population quickly. It is no accident that 1000s of cave-art sites have been discovered in 100s of countries (Blesser and Salter, 2009), indicating that a new cognitive capacity spread in the human population rapidly. In southern Africa alone, there are perhaps over a million cave-art images (Coulson and Campbell, 2001).^12^ But *H. sapiens* migrated out of Africa into the Eurasian continent some 60,000 years ago (Henn et al., 2012a). It is believed that by that time, the species already had its full modern capacity for symbolic thinking, including language (Henn et al., 2012b). Therefore, CMIT as exemplified by cave art must have started in Africa before the migration into the Eurasian continent. We turn to this topic below.^13^

## San Rock Art

The rock art of the San people constitutes evidence that rock art with CMIT properties may have existed in Africa prior to the migration of humans from the continent.

Anatomically modern humans appeared in central Africa 200,000 years ago. Gronau et al. (2011) carried out whole-genome sequencing on six individuals from different regions of the world: a European, a Yoruban, a Han Chinese, a Korean, a Bantu, and a San. The research shows that the San population was the first to split from other populations, and this occurred 108,000–157,000 years ago. They moved to southwest Africa, where they continue to reside to the present. Gronau et al. (2011) found that the Eurasian population diverged from the rest 38,000–64,000 years ago, which marks the time that *H. sapiens* began to migrate out of Africa.^14^

Along with the genetic evidence for the San’s early divergence from other human populations, there is also linguistic evidence. The San’s languages belong to the Khoisan family. All biologically Khoisan groups speak a language with phonemic clicks (Güldemann and Stoneking, 2008), which are consonants with a distinctive popping sound. Khoisan can be seen as one of the only language families in the world with clicks (Huijbregts, 2017). The only other language family is Bantu, but only in areas of contact and intermarriage with Khoisan populations, indicating the borrowing of consonantal clicks into Bantu languages (Herbert, 2002; Maddieson, 2003; Sands abd Güldemann, 2009).^15^ As Huijbregts notes, this suggests that the San, once they split off from the rest of the human population, stayed relatively isolated, something also supported by the genetic research (Gronau et al., 2011). This means that anything we find in the population may very well have been there to begin with, possibly even before they split from other human populations.

The San produced rock art that has been dated as far back as 70,000 years ago (Thackeray, 2005). The rocks were decorated because it was believed that a spirit world existed beneath the surface (Lewis-Williams and Dowson, 1990). We find this type of rock art in other regions of the world as well, typically those with an animistic tradition (Bahn and Fossati, 1995, 2003). The idea of a spirit world behind the surface of the rock could come from the acoustic property of echo: an acoustic signal is detected despite the absence of a direct source for it at the point of the sound.

There are two important points about San rock art and its relationship to symbolic thinking. First, the fact that some of the rock art predates the migration out of Africa gives credence to the hypothesis that CMIT is an example of the expression of symbolic thinking and even a factor enhancing this capacity’s spread throughout the human population. The second point relates to the apparent relative isolation of the San population from others in Africa and beyond, as indicated by the unique existence of clicks and by the genetics of the San population. The point Huijbregts (2017) makes is that the seeds of human language must have been there prior to the first genetic split, ∼125,000 years ago. This is because other populations developed a language as well. So, some cognitive property that preceded the development of language existed prior to the first human lineagesplit. If we assume that this cognitive property included symbolic thinking, the cognitive underpinnings underlying CMIT had taken root earlier than the split of the San population from the rest.^16^

## Cave and Rock Art and Human Language

Cave and rock art in general and language have a number of striking similarities:

• occur on every major continent
• possibly appeared about the same time, predating the migration out of Africa
• spread from Africa to all other parts of the world roughly at the same time
• are used for communication
• express actions, states, objects, and modification
• externalize internal mental states.

The first three points relate to the observation that artistic artifacts, either deep in caves or closer to the surface, occur on every continent occupied by modern humans (Bahn and Fossati, 1995, 2003), and the oldest occur in Africa (Henshilwood et al., 2002). The fourth point has to do with the function of art and of language: both are used for communication.^17^ The fifth point has to do with the content of artworks and of language: both may indicate actions (i.e., predicates), objects (i.e., nouns), and modification (i.e., adjectives).^18^ The final point, that art and language are external symbolic forms of internal mental states, is an obvious one, and it also may connect the two to genetic studies. The *FOXP2* gene is implicated for speech in humans and for other externalized communication forms in mice (Groszer et al., 2008; Castellucci et al., 2016; Chabout et al., 2016) and songbirds (Haesler et al., 2007), but only modern humans have art and language. Is there a genetic basis for this? *FOXP2* underwent change in modern humans (Vargha-Khadem et al., 1995; Lai et al., 2001) that affected a binding site for the transcription factor POU3F2 (Maricic et al., 2013; Huijbregts, 2017). The POU3F2 variant only occurs in modern humans, being absent from Neanderthals and Denisovans. As Huijbregts (2017) notes, this change could be seen as leading to the acquisition of full speech. Given the similarity with art, we can speculate with Huijbregts that a similar genetic change may have given rise to the multi-modal art that occurred all over the world alongside language.

## Concluding Remarks

The symbolic thinking that developed in humans led to rapid technological innovation, sophisticated visual arts, and language. This newly formed cognitive capacity may have had another, unexpected result. After continuously growing in size over the span of the Pleistocene, our brain has contracted in size by 13% in the past 20,000 years or so (Hawks, 2011 and references therein). One possible explanation is that the symbolic thinking that developed in modern humans led to a fundamentally different way to compute data, one that extracts only the essence required for abstract representation instead of computing the entire set of incoming raw data (Tattersall, 2017). Our brain membrane is metabolically expensive, so the newly formed algorithm that requires less data led to shedding of the unneeded membrane, resulting in brain diminution in recent evolutionary time. Our proposal is that the symbolic thinking pervasive in humans that led to brain diminution is exemplified, and was even enhanced, by the CMIT that we see in the cave and rock art of Africa and elsewhere in the world and by the development of language. Thus, contrary to Wallace, the development of the arts gave the modern humans a powerful evolutionary advantage.

## Author Contributions

All authors listed have made a substantial, direct and intellectual contribution to the work, and approved it for publication.

## Conflict of Interest Statement

The authors declare that the research was conducted in the absence of any commercial or financial relationships that could be construed as a potential conflict of interest.

## Footnotes

1 We understand symbolic thinking as an internalized abstract mode of reasoning capable of combining isolated symbolic representations into an array of structured meaningful expressions. Although many aspects of human symbolic abilities ought to be traced back from different animal species to some common ancestor (Cheney and Seyfarth, 1990; Zuberbühler et al., 1999; Hauser, 2001; Spelke, 2003; Carruthers, 2006; Hurford, 2007, among others), none of these aspects depend on the combinatorial mental algorithm responsible for giving rise to the distinctively human symbolic mode of thinking (Deacon, 1997; Hauser et al., 2002; Spelke, 2003; Hauser, 2009; Searle, 2009).

2 These marine shells were presumably pierced as part of a symbolic ornamentation system (Henshilwood et al., 2004). Body ornamentation may have different interpretations in different human societies, but all such artifacts are eminently symbolic (Vanhaeren, 2005; Vanhaeren and d’Errico, 2006; Kuhn and Stiner, 2007) and an archeological proxy for the presence of a symbolically mediated behavior (d’Errico and Vanhaeren, 2009).

3 See also d’Errico et al. (2001), Henshilwood and Dubreuil (2009), Mourre et al. (2010).

4 It is also worth mentioning that although apes can understand referential symbols (Rumbaugh and Washburn, 2003; Tomasello et al., 2005) and develop cultural traditions (Whiten, 2005), they do not spontaneously create symbolic systems in the wild (Deacon, 1997; d’Errico and Vanhaeren, 2009).

5 There are a number of approaches to the evolutionary development of the high cognitive capacity in humans. See, for example, Heyes and Frith (2014) for a summary of some of these approaches. Some postulate that human cognition developed gradually over a long period, starting as early as before the Pleistocene. While we do not doubt that the basic cognitive capacity existed in early hominids, we believe that the big “push” that led to the unique and pervasive symbolic thinking of modern humans developed much later in evolution.

6 The acoustic-visual correlation is widespread and it was found in the caves of France (viz., Le Portel, Niaux, Isturitz, Arcy-sur-Cure, Font-de-Gaume, Lascaux, Bernifal, among others; see Reznikoff and Dauvois, 1988; Waller, 1993b; Reznikoff, 1995, 2006; Dauvois, 1996, 1999, 2005), in the caves of Spain (viz., La Garma, El Castillo, La Pasiega, Las Chimeneas, Tito Bustillo; see Fazenda et al., 2017), and on two other continents (Waller, 1993a). Such diversity indicates that the correlation is not random.

7 Similar patterns were attested in other cave sites in France, such as Les Combarelles, Bernifal, Bara-Bahau, and Cougnac (see Waller, 1993b, for a detailed list).

8 In a recent article, Fazenda et al. (2017) report on highly sophisticated acoustic tests together with statistical analysis in five caves in Northern Spain (viz., La Garma, Las Chimeneas, La Pasiega, El Catillo, and Tito Bustillo). They found that lines and dots are likely to occur in areas of low reverberation, while motifs of animals and other such depictions are likely to occur in areas with higher reverberation.

9 A reviewer observes that this image may have a hierarchical structure, which could possibly connect cave art to language.

10 This perspective differs from other approaches to language evolution, such as Deacon’s (1997, 2012), which proposes that the critical evolutionary threshold for the development of human language was the emergence of symbolic reference, allegedly absent in non-human animals. Many recent works on animal cognition suggest that the symbolic richness we find in the human species is attested in non-human animals (Cheney and Seyfarth, 1990; Zuberbühler et al., 1999; Hauser, 2001; Spelke, 2003; Carruthers, 2006; Hurford, 2007; Carey, 2009; among others). If this turns out to be correct, then it is plausible to claim, contrary to Deacon, that the emergence of a system of thought, rather than a semiotic innovation, was the key evolutionary ingredient that led to the emergence of human language. As Searle (2009) points out, it was syntax —i.e., the generative engine underlying such system of thought— that organized semantics, producing a new kind of logical representation in humans. As often speculated, the complexity of this system of thought did not emerge incrementally, rather it was likely full-fledged from its very beginning, even though it was not immediately externalized (Di Sciullo, 2014; Nóbrega and Miyagawa, 2015).

11 It is not evident who in society was creating these art forms, but there are certain characteristics about the placement of the art and its content that lend to indirect claims about artists and their purposes. For instance, the rock art of Kugpal, south India, is located in an area of very difficult access, which would preclude the engagement of large groups. According to Boivin (2004, p. 45), this implies that “the activities surrounding rock art may have been limited to a particular sector of the society,” since not all members could equally participate in art production and consumption. The same sense of exclusivity emerges when we consider the shamanistic elements found in painted caves and rocks. Such features tend to support an association of these art forms with sacred rituals (see Lewis-Williams and Dowson, 1990; Lewis-Williams, 1991, 1997; Whitley, 1992, 1994, 1998; Clottes and Lewis-Williams, 1998; Ryan, 1999; Winkelman, 2002; Reznikoff, 2014), which consequently leads to the speculation that the artist could have been him/herself a shaman. Setting aside these speculations regarding authorship, it is reasonable to claim that the individuals who were endowed with the ability to translate symbolic thinking into sensory stimuli may have had reproductive advantages.

12 It is worth mentioning that the best-known examples of cave art are found in Europe, dating back 40,000 years to the Upper Paleolithic period (Pike et al., 2012).

13 It is important to highlight that the CMIT hypothesis does not rule out that other, parallel strategies for externalizing symbolic thinking may have been employed by early modern humans, such as manual gestures (Corballis, 2003, 2009, 2013), pantomimes and imitation (Arbib, 2002, 2009, 2012), and even sign language (Armstrong and Wilcox, 2007). Nevertheless, since we have to rely on indirect evidence to tentatively re-create the environment in which a symbolic mode of reasoning was externalized, the safest approach is to elaborate scenarios that can be empirically reconstructed. Such is the case with the discoveries in archeoacoustics: these can be recreated by means of, for instance, ambisonics technique, which can be applied “to identify the likely use of echolocation among societies for which no ethnographic information remains” (Mattioli et al., 2017, p. 12).

14 In a separate study, Veeramah et al. (2012) resequenced samples from four populations in Africa —San, Eastern Pygmies, Western Pygmies, and non-Pygmy Niger-Kordofanian speakers— at 40 non-genic (∼2 kb) regions and came to essentially the same conclusion, that the San population diverged first, approximately 110,000 years ago.

15 A piece of evidence for the borrowing of consonantal clicks into Bantu languages is the absence of clicks in Bantu languages outside the area where Khoisan languages are found (Pakendorf et al., 2017). Furthermore, genetic analyses of populations indicate that there was a prehistoric contact between Khoisan and Bantu-speaking populations. For instance, Barbieri et al. (2013) undertook an extensive genetic study of the Bantu population in order to elucidate the nature of the borrowing. Their analysis favors “admixture in the maternal line between some of the Bantu groups from Zambia and Khoisan-speaking populations,” suggesting that the borrowing took place through “incorporation of Khoisan women” into the Bantu population; a conclusion endorsed by Pakendorf et al. (2017).

16 A reviewer brought to our attention that the strong identifications between the San people and the eland, a type of antelope, may also express a mental representation in CMIT terms. According to Opoku (2006, p. 356), 77% of the San rock arts involve antelopes. The importance of the antelope —more specifically, the eland— in San mythology can be noticed in their ritual behaviors, whereby the young San boys and girls virtually identify themselves with elands. As the reviewer points out “the visual stimuli of the eland is transformed into the kinetic gestures of the ritual” by the community, which may indicate that the artist who created the paintings “need not be the only source of the innovation in externalized expression although clearly he/she is the only one who left evidence of his/her cognitive ability.”

17 The communicative function of art can be inferred from the finding that cave paintings may have played a role in Paleolithic man’s religious rituals (Reznikoff, 2014).

18 We can even surmise that lexical categories such as verb, noun, and adjective may have been expressed in many of these artistic representations.
